# A Treatise on Midwifery, &c.

**Published:** 1857-11

**Authors:** 


ART. XII.—*A Theoretical and Practical Treatise on Midwifery, including the Diseases of Pregnancy and Parturition, and the Attentions required by the Child from Birth to the Period of Weaning.* By P. CAZEAU, Adj. Prof. in the Faculty of Medicine of Paris. Second American Edition, translated from the Fifth French Edition, by WM.

R. BULLOCK, M.D., with 140 Illustrations. Philadelphia: Lindsay & Blakiston. 1857.

WE have received the above large volume from the publishers, and take pleasure in welcoming M. Cazeaux to a renewed American acquaintance. In our next number, we hope to present our readers with an extended analysis of the work; at present, we can do no more than thank the publishers for the privilege, a privilege which the handsome appearance of the book would of itself shame a reviewer from abusing, were better motives wanting.

